# Environmental health policies for women’s, children’s and adolescents’ health

**DOI:** 10.2471/BLT.16.171736

**Published:** 2017-07-24

**Authors:** Maria Neira, Elaine Fletcher, Marie Noel Brune-Drisse, Michaela Pfeiffer, Heather Adair-Rohani, Carlos Dora

**Affiliations:** a Department of Public Health, Environmental and Social Determinants of Health, World Health Organization, avenue Appia 20, 1211 Geneva 27, Switzerland.

Environmental health risks especially affect women and children, because they are more vulnerable socially and because exposures to environmental contaminants create greater risks for children’s developing bodies and cognitive functions. According to the 2016 World Health Organization (WHO) estimates, modifiable environmental risk factors cause about 1.7 million deaths in children younger than five years and 12.6 million total deaths every year.^1^

Although the *Global strategy for women’s, children’s and adolescents’ health (2016–2030)*^2^ was launched during the United Nations Sustainable Development Summit 2015, governments rarely recognize the sustainable development agenda as a transformative factor for health. The sustainable development goals (SDGs) offer opportunities for countries to create healthier environments for women, children and adolescents.

This paper explores how the SDGs can be used to reduce environmental health risks and enhance the health of women, children and adolescents. In particular, we focus on drivers for urbanization and sustainable development (e.g. transport, housing, urban design and energy provision) that can advance the global strategy, but have not traditionally been a focus of health policy-making. We frame the discussion around the three pillars of the global strategy: survive, thrive and transform, while recognizing the inevitable overlap between these objectives.

## Survive

Since women and children are especially affected by the environment, intersectoral interventions that reduce environmental risks will improve early childhood survival as well as reducing risks of premature death throughout the life-course.

For instance, household air pollution from dirty fuels and inefficient cookstove technologies was estimated to have caused around 4 million premature deaths in 2012 and was responsible for more than half of deaths due to childhood pneumonia.^3^ Among women, indoor exposures to household cookstove smoke were estimated to cause 34% (452 548/1 336 601) of chronic obstructive pulmonary disease deaths, 21% (732 937/3 476 815) of stroke deaths, 19% (93 537/489 390) of lung cancer deaths and 14% (479 478/3 425 835) of ischaemic heart disease deaths in 2012.^4^^,^^5^

Improving access to reliable electricity and clean water in health-care facilities can also help reduce maternal and newborn mortality, as such infrastructure is a critical determinant of quality of care.^6^ A review of health-care facilities in 11 sub-Saharan African countries showed that an average of 26% of facilities had no electricity whatsoever.^7^ Another review of 54 low- and middle-income countries found that 38% (25 118/66 101) of health facilities lack a clean drinking water source.^8^ Ensuring that health-care facilities have access to power and water is a minimum requirement for attracting women to facilities and guaranteeing quality services for safe childbirth.

## Thrive

Housing and energy sector interventions that promote the transition to cleaner fuels and technologies for domestic cooking, heating and lighting can not only reduce deaths but improve the health of the 3 billion people worldwide who are reliant upon inefficient and polluting cookstoves.

For this reason, the monitoring framework of the *Global strategy for women’s, children’s and adolescents’ health* *(2016–2030)* explicitly tracks an indicator for “primary reliance on clean fuels and technologies” in households as part of its thrive pillar.^9^ Examples of cleaner fuels and technologies include liquefied petroleum gas, biogas, ethanol and electricity including photovoltaic solar-power for lighting. Improving access to clean fuels and technologies can also reduce the burden of childhood burns and poisonings due to the use of kerosene for cooking and lighting.

While most of the estimated 3 million deaths annually from outdoor ambient air pollution are among adult populations, reducing such pollution exposures are also critical to improving children’s health and development across the life-course.^10^

Currently, more than 92% of the world’s urban population is exposed to average annual air pollution concentrations above WHO guideline levels for fine particulate matter PM_2.5_ – that is, particles smaller than 2.5 μm in diameter. In developing cities, concentrations may be many times above guideline levels,^11^ and children in these cities experience chronic exposure to high levels of PM_2.5_ and ground-level ozone.^12^ These chronic exposures reduce children’s lung function at critical developmental stages, which increase the risk for chronic respiratory illnesses including asthma, as well as cardiovascular disease, stroke and cancers later in life.^13^

Air pollution also affects the health of high-income populations. For example, 67% (1043/1546) of the high-income European cities monitored by WHO fail to meet WHO guidelines levels for PM_2.5_. A study of air pollution-related health impacts in 25 European cities, totalling nearly 39 million inhabitants, showed that in cities with air pollution above the WHO guideline for annual mean PM_2.5_, achieving compliance would add up to 22 (2-22) more months of life expectancy at the age of 30 years, as well as generating some 31 billion euros annually in health and related savings overall.^14^

In many low- and middle-income cities, the lack of efficient public transport infrastructure stimulates reliance upon private transport modes and further exacerbates air pollution. People lacking private vehicle access experience an increased risk of traffic injury due to the lack of safe pedestrian and cycling spaces.^15^ In addition, the lack of safe outdoor spaces for children to play and enjoy physical activity contributes to sedentary lifestyles for rich and poor alike, contributing to childhood obesity.^16^

Air pollution is just one of the routes by which environmental contaminants affect children’s development, both *in utero* and in the early years of life. Estimates show that about 200 million children worldwide fail to reach their full potential due to, among others, toxic exposures to lead and mercury, either directly or through water, foods and waste.^17^^,^^18^ Both mercury and lead negatively affect the nervous system of the developing fetus and slow the cognitive development of young children.

While noncommunicable diseases now constitute two-thirds of the environmentally-related health burden,^1^ controlling environmentally-related infectious diseases also remains a challenge. Infectious diseases continue to present significant risks for the unborn child and for young children whose adaptive immune systems are under-developed. For example, unplanned urbanization, often characterized by poor housing and deficient environmental services for water, waste and sanitation, is a factor in vector-borne disease transmission. Such urbanization, as well as changing climate patterns, has been recognized as a driver promoting the proliferation of *Aedes aegypti*, the primary vector for dengue and Zika viruses.^19^ The Zika virus can cause congenital Zika virus syndrome, including microcephaly.^20^ Urban planning that reduces vector breeding sites and improves house-screening measures, may help protect women and children from bites and reduce transmission risks of vector-borne diseases.^21^

## Transform

The global strategy aims for a holistic approach by supporting strategies that reduce avoidable risks to women’s, children’s and adolescents’ health. Interventions to transform health-care delivery, social and gender equity are core themes. However, as part of a holistic approach, the strategy also needs to prioritize environmental health interventions in cities as well as rural areas. Aligning the global strategy more clearly with SDGs beyond the health-related SDG 3 and gender-equality-related SDG 5 helps promote a more integrated view of health and global development. 

For instance, SDG 11.2 calls for improving safe, accessible public transport, “with special attention to the needs of those in vulnerable situations, women, children, persons with disabilities and older persons.” Achievement of this target can reduce traffic injury risks for these groups, as well as improving mobility.

Similarly, greater alignment between strategies aimed at reaching SDG 3 targets on maternal and newborn survival with goals for water and sanitation (SDG 6), sustainable energy (SDG 7), and climate change (SDG 13) opens possibilities to address deficiencies in health-systems infrastructure and constraints in health-care delivery that limit quality of care.

Reducing mortality from air pollution exposures is referenced as a progress indicator in SDG 3. The preconditions for reducing such mortality – such as increased access to clean household fuels and technologies and reduced urban ambient air pollution exposures, are indicators of the SDGs for energy (7.1.2) and cities (11.6.2), respectively. These indicators reflect the inextricable linkage between environmental risk and health effect.

Numerous recent health sector resolutions pave the way for closer alignment between women’s and children’s health and economic development goals. For instance the 2015 World Health Assembly (WHA) resolution: *Health and the environment: addressing the health impact of air pollution*^22^ explicitly recognized the impacts of air pollution on vulnerable groups, calling for greater leadership by the health sector.

The Minamata Convention on reducing non-essential uses of mercury was also recently affirmed by a WHA resolution.^23^ The Global Alliance to Eliminate Lead Paint, jointly led by WHO and the United Nations Environment Programme has led to legally-binding controls on the production, import, export, sale and use of lead paint in 64 countries.^24^ The United Nations Sustainable Energy for All initiative empowers governments to consider and to monitor household air pollution, as well as health sector access to clean energy sources.^25^

Awareness-raising is also important for motivating the public and politicians to tackle environment and health risks. The global BreatheLife campaign (available at: www.breathelife2030.org), which addresses public health and climate change goals simultaneously, is promoting awareness about air pollution by providing a platform where cities can commit to WHO air quality goals, and share best practices and their progress. The platform is also educating the general public about air pollution and how they can take actions.^26^

These international commitments and initiatives pave the way for national and city governments to act more assertively to revise their development strategies related to transport, housing and energy with environmental health priorities in mind. Health ministries need to be poised as leaders in this effort. Ministries need the know-how and capacity to stimulate intersectoral collaborations; explain to other sectors the connections between environmental risks and health; and provide evidence about the best interventions. Health ministries can also monitor development trends, such as women’s improved access to clean energy in households and health facilities, or in cities, access to safe and sustainable transport, in relation to mortality and morbidity, as per the relevant SDG targets and indicators.

In India, for instance, an inter-ministerial commission has been formed to address air pollution as a health issue. National and regional energy initiatives can address environmental health risks at household level. The Lighting a Billion Lives© initiative,^27^ offers such an example of a non-health sector intervention that benefits health. The initiative which is active in about 24 countries across South-East Asia, replaces inefficient and harmful lightning and cookstoves with efficient, affordable and reliable energy systems.

At the city level, urban transport and design strategies can address risks from ambient air pollution and traffic injuries, as well as fostering safer outdoor spaces where children and families can walk to school, play safely and be active. More walkable and transit-friendly cities can benefit women’s mobility, provide greater access to jobs and education, and improve gender equity.^12^^,^^15^

Too often, choices regarding economic development strategies boil down to cost−benefit assessment models that fail to consider long-term health impacts and health externalities. In the SDG era, policies and investments in urban design, transport, housing and energy need to be assessed so that the health costs of air pollution, dirty energy, unhealthy housing and traffic injury risks, are taken into account.

Indeed, the evidence is that more sustainable urban development investments create more long-term benefits to society, because of people’s improved health status, which in turn improves economic productivity and reduces health costs to society as well as catastrophic health-care costs to households.

Using the SDGs to make cities healthier, promote cleaner air and water, and ensure clean, reliable energy access in climate resilient health-care facilities will reduce pollution-related deaths and illnesses, particularly among women and children. Therefore, interventions addressing environmental health risks should be integral to the vision of the global strategy.
